# Engineered *Bacillus subtilis* regulates intestinal barrier in ETEC K88‐infected piglets and modulates miR‐1250‐5p/NFκB pathway in IPEC‐J2

**DOI:** 10.1002/imo2.70094

**Published:** 2026-05-11

**Authors:** Chaoyue Wen, Hong Zhang, Qiuping Guo, Yehui Duan, Sisi Chen, Mengmeng Han, Leli Wang, Le Huang, Yuankun Deng, Fengna Li, Yizhen Wang, Mingliang Jin

**Affiliations:** ^1^ National Engineering Research Center for Green Feed and Healthy Breeding; Key Laboratory of Molecular Animal Nutrition, Ministry of Education, Institute of Feed Science, College of Animal Sciences Zhejiang University Hangzhou China; ^2^ Key Laboratory of Animal Nutrition and Feed Science (Eastern of China) Ministry of Agriculture and Rural Affairs; Zhejiang Key Laboratory of Nutrition and Breeding for High‐Quality Animal Products; Zhejiang University Hangzhou China; ^3^ College of Food Science and Technology Hunan Agricultural University Changsha China; ^4^ Hunan Provincial Key Laboratory of Animal Nutritional Physiology and Metabolic Process, CAS Key Laboratory of Agro‐Ecological Processes in Subtropical Region, Institute of Subtropical Agriculture Chinese Academy of Sciences Changsha China; ^5^ National Engineering Laboratory for Pollution Control and Waste Utilization in Livestock and Poultry Production, Institute of Subtropical Agriculture Chinese Academy of Sciences Changsha China; ^6^ College of Advanced Agricultural Science University of Chinese Academy of Sciences Beijing China; ^7^ College of Animal Science and Technology Hunan Agricultural University Changsha China

**Keywords:** antimicrobial peptide, barrier function, engineered Bacillus subtilis, immune response, weaned piglet

## Abstract

The substantially increased prevalence of diarrhea in infants and livestock induced by enterotoxigenic *Escherichia coli* (ETEC) infection threatens the intestinal health of humans and livestock. This study investigated an engineered *Bacillus subtilis* WB800_KR32 strain expressing antimicrobial peptide KR32 (yielding 2.24 μg/mL with peak activity at 33 h fermentation) for its preventive efficacy against ETEC K88‐induced diarrhea in a piglet model. Oral administration of WB800_KR32 could significantly improve intestinal morphology and barrier function, particularly in the ileum, reduce *Escherichia* relative abundance, and enhance repair and metabolism‐related pathways while increasing serum antioxidants such as glutathione. Mechanistically, WB800_KR32 significantly decreased novel‐ssc‐miR‐1250‐5p expression and its predicted target, nuclear factor kappa‐B (NFκB) p65 (*p* < 0.05). In vitro, 40% fermented WB800_KR32 supernatant reduced novel‐ssc‐miR‐1250‐5p expression, improved tight junction function, decreased inflammatory response, and reduced NFκB p65 phosphorylation in ETEC K88‐infected IPEC‐J2 cells (*p* < 0.05). These findings suggested that engineered *Bacillus subtilis* WB800_KR32 provides a dual‐action therapeutic platform combining targeted antimicrobial delivery with host immunomodulation, offering a promising translational strategy for preventing ETEC K88‐induced diarrhea in humans and livestock.

## Highlights

- WB800_KR32 offers a dual‐action therapeutic platform that combines antimicrobial delivery with host immunomodulation to prevent ETEC K88‐induced diarrhea.
- WB800_KR32 significantly improved intestinal morphology and barrier function.
- WB800_KR32 decreased novel‐ssc‐miR‐1250‐5p expression and its predicted target NFκB p65.

## Graphical abstract

Upon enterotoxigenic *Escherichia coli* (ETEC) K88 infection, intestinal barrier function is disrupted with compromised tight junctions, accompanied by an exacerbated inflammatory response, proliferation of pathogenic Escherichia, and depletion of beneficial Lactobacillus paralimentarius. In parallel, miR‐1250‐5p expression is elevated, and both NF‐κB mRNA and phosphorylated p65 protein levels are increased. With WB800‐KR32 pretreatment followed by ETEC K88 challenge, KR32 antimicrobial peptides are secreted to directly inhibit Escherichia, intestinal barrier integrity is restored with reduced inflammation, the microbiota is reshaped with increased *Lactobacillus paralimentarius*, decreased Escherichia, and altered abundances of *Romboutsia sedimentorum* and *Clostridium chartatabidum*, miR‐1250‐5p expression is downregulated, and NF‐κB signaling is suppressed, as evidenced by reduced NF‐κB mRNA levels and diminished p65 phosphorylation. Central Mechanism: The engineered bacterium employs a two‐pronged strategy: modulating the miR‐1250‐5p/NF‐κB pathway and delivering KR32 antimicrobial peptide to improve gut microbiota composition, strengthen intestinal barrier function, and mitigate ETEC K88‐induced inflammatory damage.

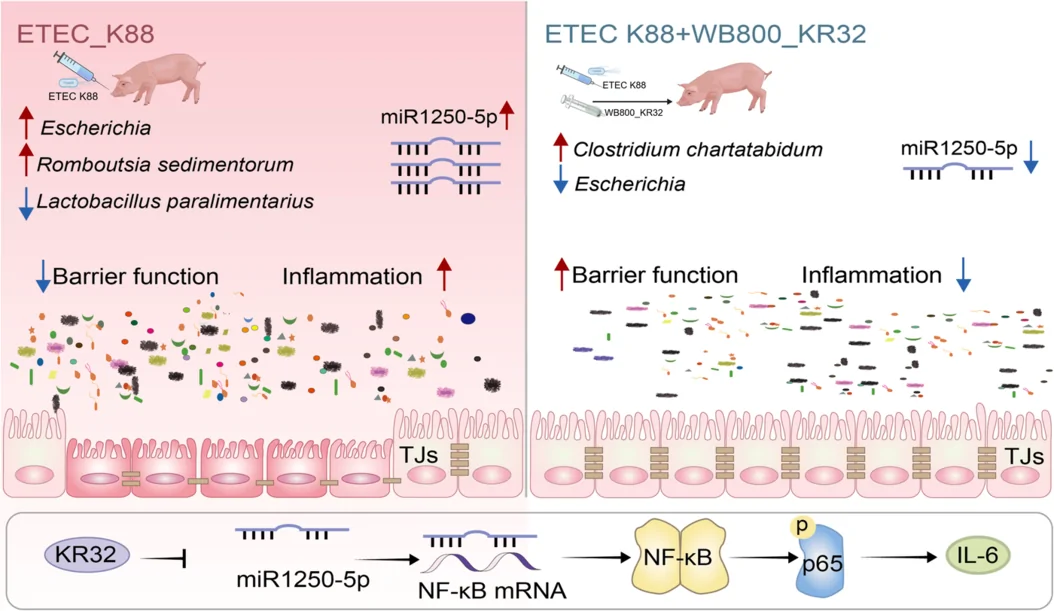

## 1 INTRODUCTION

Diarrheal disease is the second leading cause of death in children less than 5 years old, and almost 525,000 deaths in children in 2017 (World Health Organization. Diarrheal disease (updated May 2017)). Weaning is the most critical period in pig production, frequently associated with severe enteric infection [1]. Enterotoxigenic *Escherichia coli* (ETEC) is the main cause and also a common cause of weaned piglets. Overuse and inappropriate consumption of antibiotics have generated serious public health concerns due to the rapid emergence of multidrug‐resistant bacteria [2]. The risk of diarrhea caused by ETEC is hard to effectively control [3] and usually leads to great economic loss in the swine industry worldwide. ETEC causes disease by colonizing the small intestine epithelial cells. The current challenge is finding new effective nonantibiotic options to reestablish gut microbial and barrier function in weaned piglets. Probiotics, essential oils, and plant extracts exhibited potential against ETEC infection in piglets [4, 5, 6]. However, their protective effects are limited. Antimicrobial peptides (AMPs), also referred to as host defense peptides, have potential therapeutic implications for varied enteric disorders, not only as antimicrobial drugs, but also as advanced product candidates for feedback regulation of cytokine response [7, 8], and a regulator of interaction between antimicrobial responses with pathogens and the intestinal microbiota [9]. Opportunely, AMPs can be rationally designed and optimized. AMP KR32, which showed high antimicrobial activity, slight hemolytic activity, and cytotoxicity, was designed based on peptide cathelicidin‐BF [10]. However, AMPs have a series of problems to be resolved, such as weak oral bioavailability, potential cytotoxicity, and immature stability [11, 12, 13], which greatly limit their application. Furthermore, the yield of natural AMPs is too low, and the price of chemical synthesis of AMPs is too high, which remains a main barrier to the commercialization of AMPs [8].

Engineered living materials to produce antimicrobial and immune‐modulating properties are a great area in synthetic biology. Because live engineered *Bacillus subtilis* delivers the targeted molecule in situ, the recombinant antimicrobial peptide does not need isolation and purification, which is a major advantage. Recent research suggested that microRNAs (miRNAs) can regulate immune response and intestinal barrier function during pathogen infection and pathophysiology [14, 15]. Pig is the ideal model for the study of human intestinal disease, for its similar physical structure and function [16]; thus, our study could provide a reference for human nutrition.

In this study, we first evaluate the antimicrobial activity and content of fermented supernatant of engineered WB800_KR32. And then evaluate the preventive effect of engineered WB800_KR32 and its supernatant on the intestinal barrier in ETEC K88‐infected piglets and IPEC‐J2 cells, respectively. And finally, we explore the potential mechanisms of miRNAs in it. Thus, the engineered WB800_KR32 has the potential to develop into a new feed additive for preventing ETEC K88‐induced diarrhea.

## 2 RESULTS

### The bioactivity and yield of engineered WB800_KR32

The expression pattern of the reporter plasmid in WB800 is shown in Figure S1. We have applied the Oxford cup method and liquid chromatography‐tandem mass spectrometry (LC/MS/MS) analyses to measure the antimicrobial activity and the content of KR32, respectively. Results showed that the supernatant of WB800‐KR32 (grown at 33 h) reached the highest anti‐*E. coli* activity (Figure 1A,B). Meanwhile, we tested the antibacterial activity of WB800, WB800‐pBE2R, and LB. Results showed that WB800‐KR32 had the highest antibacterial activity among the other groups (Figure 1C,D). The counts and acquisition time of the targeted compound (KR32) were presented in Figure 1E,F. The multiple‐reaction monitoring (MRM) conditions used for LC/MS/MS analysis of analytes are shown in Table S1. The supernatant of fermented WB800_KR32 was mixed with acetonitrile (v/v, 25%), and then dried with a nitrogen concentrator, dissolved with 25% acetonitrile, and filtered through a 0.22 μm filter. According to the peak area and concentration of standard KR32, the concentration of supernatant of WB800_KR32 was 2.24 μg/L.

**Figure 1:**
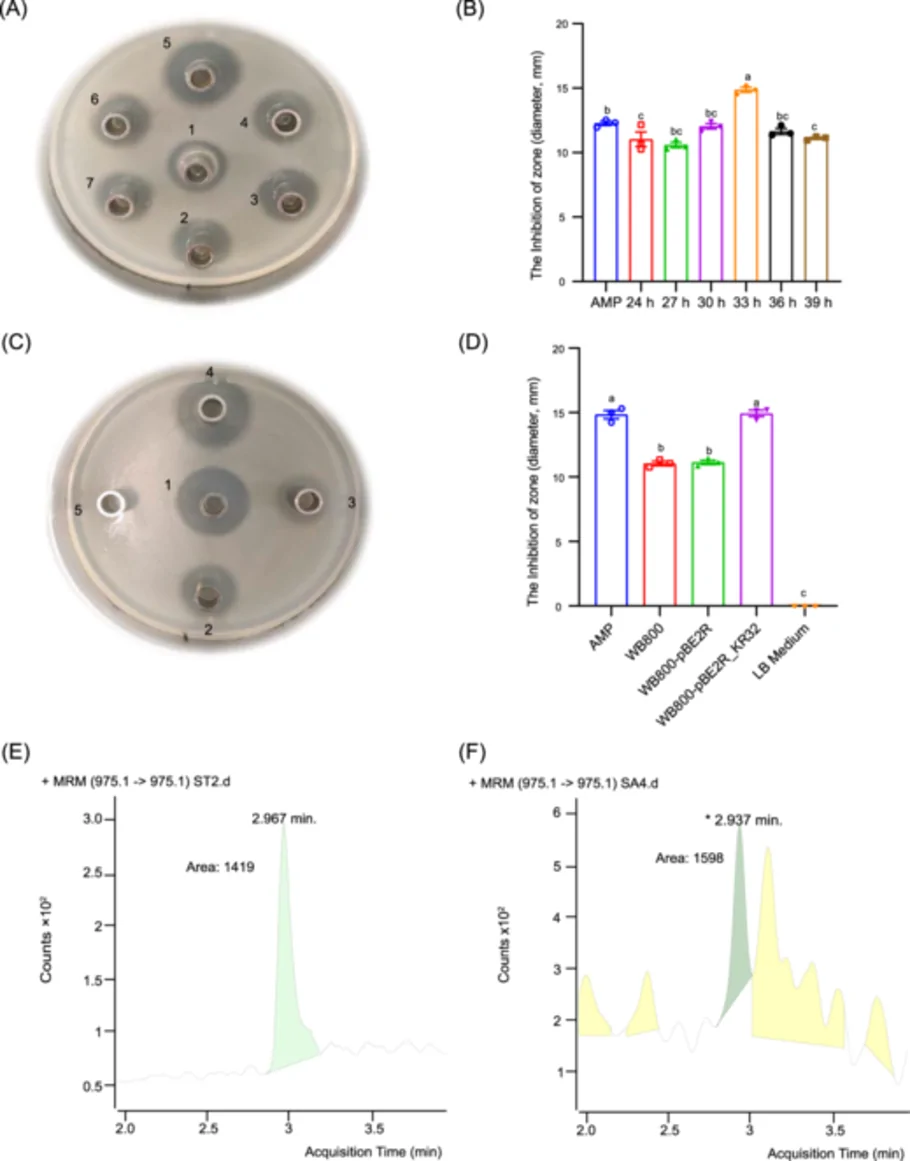
The anti‐ETEC K88 activity of supernatant of fermented WB800_KR32 and the level of KR32 in the supernatant (*n* = 4). Time‐course of antimicrobial activity against ETEC K88, representative plates (A) and quantification (B). 1: 10 μg/mL Ampicillin (AMP); engineered WB800_KR32 fermented supernatant at varied time 2: 24 h; 3: 27 h; 4: 30 h; 5: 33 h; 6: 36 h; 7: 39 h. Samples were collected at the indicated fermentation time points and tested by agar well diffusion assay (*n* = 4 biological replicates). Data represent mean ± SD of inhibition zone diameters (mm); representative plates (C) and quantification (D) showing inhibition zones of 33 h supernatant compared to standard KR32. 1: 10 μg/mL Ampicillin (AMP); fermented 33 h supernatant of 2: WB800, 3: WB800‐pBE2R, 4: WB800‐pBE2R‐KR32, 5: LB broth. LC/MS/MS of chemically synthesized KR32 standard (E) and fermented WB800_KR32 supernatant (33 h) (F) showing peak acquisition times used for quantification. Differences in (B) and (D) were evaluated using one‐way ANOVA with Duncan's multiple range test.

### Engineered WB800_KR32 improves the growth performance and intestinal barrier function in ETEC K88‐infected piglets

Before ETEC K88 infection, compared with the control (CON) group, oral WB800_KR32 had no significant effect on growth performance (Table S2). Post to ETEC K88 infection, oral WB800_KR32 and ETEC K88 infection had no significant effect on average daily feed intake; compared with the ETEC K88 group and the ETEC + WB800 group, the average daily gain (ADG) was significantly increased, the feed/gain and diarrhea score were significantly decreased in ETEC + WB800_KR32 group, while showed no significant difference with CON group (Figure 2A–D).

**Figure 2:**
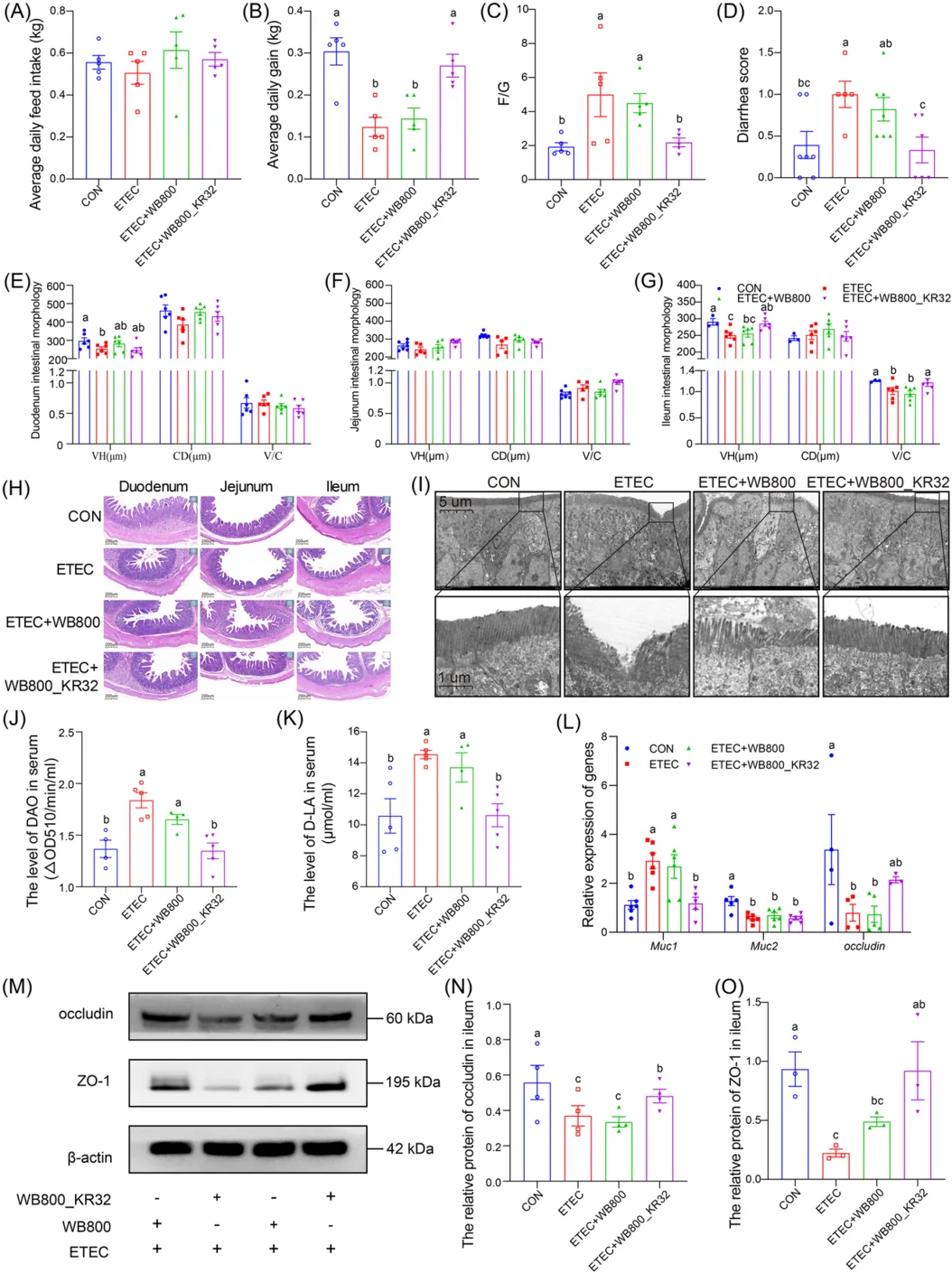
WB800_KR32 improves growth performance and intestinal barrier function in ETEC K88‐infected piglets (*n* = 7). The effect of WB800_KR32 on the growth performance: average daily feed intake (A), average daily gain (B), F/G (C) in ETEC K88 infected piglets; the effect of WB800_KR32 on diarrhea score in post ETEC K88 infected piglets (D); the effect of WB800_KR32 on intestinal morphology: duodenum (E), jejunum (F), ileum (G), the representative H&E staining of small intestine (200× magnification) (H) in ETEC K88 infected piglets; the micro‐morphology of the ileum using the TEM method (scale bar = 1 μm) (I); the serum biomarker of intestinal permeability: DAO (J) and D‐LA (K) in; the relative mRNA expression of the tight junction genes (*claudin‐1*, *occludin*, *ZO‐1*) in ileal mucosa (L); Western blot analysis of tight junction proteins: representative blots (M), the relative expression of occluding (N), the relative expression of ZO‐1(O). Data are presented as mean ± SEM; bars not sharing the same letter are significantly different (*p* < 0.05).

The results of intestinal morphology were presented in Figure 2E–H. Compared with the ETEC group, the duodenal villus height was significantly increased in the CON group. The jejunal and ileal villus heights and villus height/crypt depth were significantly increased in the ETEC + WB800_KR32 group, showing no significant difference compared with the CON group. TEM results showed that the ETEC + WB800_KR32 group had an improved microvillus compared with the ETEC group (Figure 2I).

We examined the intestinal permeability and barrier function; the results are shown in Figure 2J–K. Compared with the ETEC group, the activity of diamine oxidase (DAO) and the level of D‐lactic acid (D‐LA) in serum were significantly decreased and showed no significant difference with the CON group. Compared with the CON group, the gene expression of *occludin* and *Muc2* was significantly down‐regulated, and *Muc1* was significantly up‐regulated in the ETEC group, while the gene expression of *Muc1* showed no significant difference with the ETEC + WB800_KR32 group (Figure 2L). Compared with the ETEC group, the relative protein expression of occludin and ZO‐1 was significantly up‐regulated in the ETEC + WB800_KR32 group (Figure 2M–O). The cytokines in serum and intestinal mucosae are presented in Table S3.

### Engineered WB800_KR32 modulates ileal microbiota in ETEC K88‐infected piglets

We analyzed the microbial composition of ileal content. The α‐diversity of ileal microbiota is shown in Table S4. The distance of NMDS based on Bray–Curtis showed that the microbial community of WB800_KR32 treatment and ETEC infection could be significantly distinguished (Figure S2A). The analysis of principal coordinates analysis (PCoA) based on unweighted‐unifrac revealed distinct clustering of microbiota composition for each treatment (*p* = 3e‐04, *R* ^2^ = 0.22, Figure 3A). The results of partial least squares discrimination analysis (PLS‐DA) were similar to PCoA (Figure 3B). The relative abundance of Escherichia is 3.81%, 33.67%, 28.13%, and 2.15% in CON, ETEC, ETEC + WB800, and ETEC + WB800_KR32, respectively (Figures S2B, 3C). *T*‐test of differentially abundant taxa revealed that, compared with the control, the ETEC group exhibited a marked reduction in the relative abundance of *Lactobacillus paralimentarius* (OTU36) and a significant increase in *Romboutsia sedimentorum* (OTU1) (*p* < 0.05). (Figure S2B) Conversely, relative to the ETEC group, the ETEC_KR32 group displayed a pronounced elevation in *Clostridium chartatabidum* (OTU7) and a significant decline in *Escherichia* (OTU2) (*p* < 0.05). (Figure 3C). The heatmap of β‐diversity is shown in Figure 3D, which suggests that the repeatability within group is good while the variation between groups is significantly different. The overall microbial composition of each group differed at the species level. We found that the predicted functions of microbiota were disturbed by ETEC K88 infection and reversed by WB800_KR32 treatment. Specifically, compared with the CON group, the pathways of Infectious disease: Parasitic, Infectious disease: Bacterial, and signal transduction were enriched in the ETEC group (Figure S2C). Compared with the ETEC group, the pathways of amino acid metabolism, cell growth and death, replication, and repair were enriched in the ETEC + WB800_KR32 group (Figure S2D). Correlation analyses between pathological injury indices and differential microbiota revealed that *Romboutsia sedimentorum* abundance was negatively correlated with ileal mucosal IL‐6 (*ρ* = −0.49, *p* = 0.009, [−0.72, −0.14]). *Lactobacillus paralimentarius* abundance showed a positive correlation with average daily weight gain of piglets (*ρ* = 0.47, *p* = 0.012, [0.16, 0.72]), whereas *Escherichia* abundance was positively associated with both diarrhea score (*ρ* = 0.46, *p* = 0.015, [0.13, 0.74]) and ileal mucosal IL‐6 levels (*ρ* = 0.38, *p* = 0.047, [−0.01, 0.68]) (Figure 3E).

**Figure 3:**
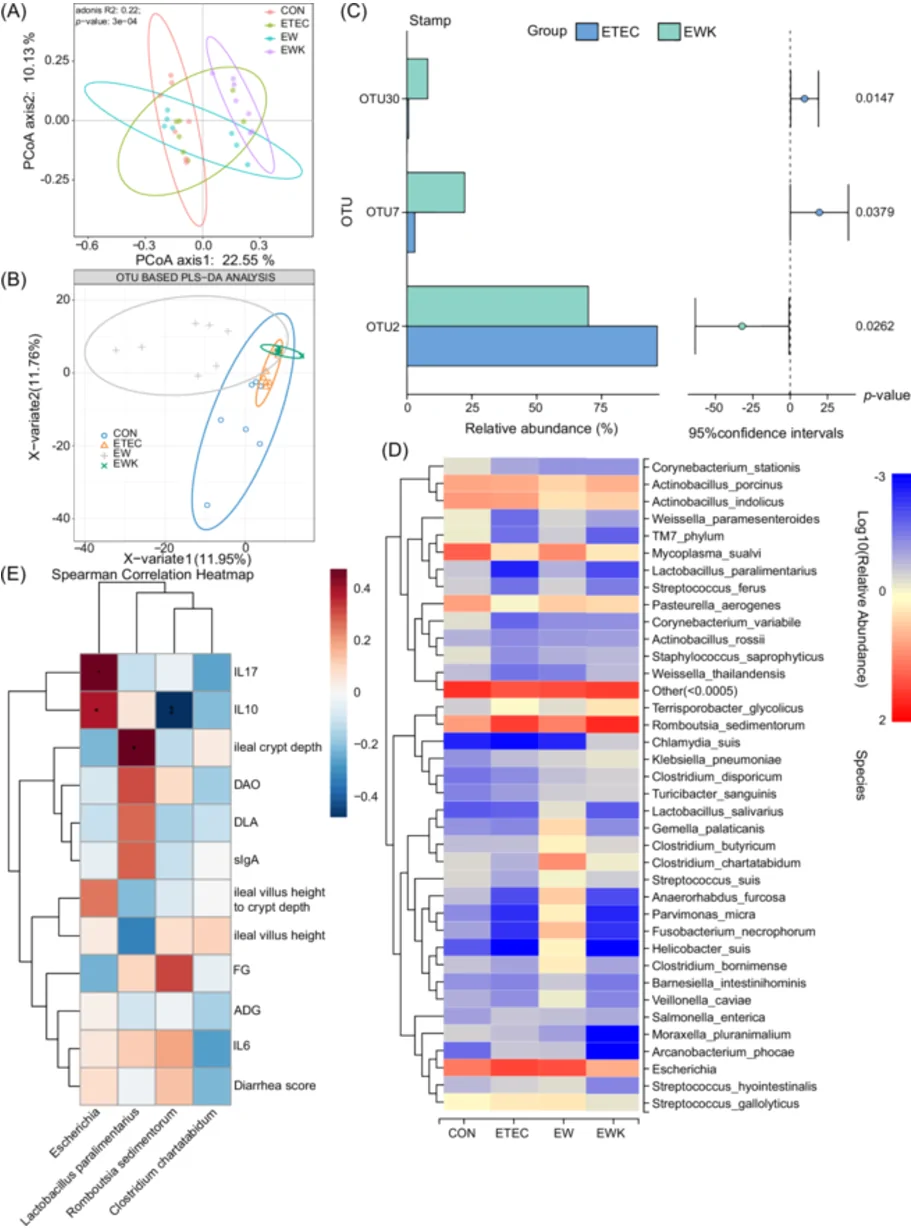
WB800_KR32 induces a major reshaping of the ileal microbiota in enterotoxigenic *Escherichia coli* (ETEC) K88‐infected piglets (*n* = 7). Analysis of the PCoA plots based on the unweighted‐unifrac distance metric; each point represents one piglet (A); the effect of WB800_KR32 on PLS‐DA score plot showing group separation (B); *t*‐test and FDR correlation (BH/*q*‐value) on species‐level differential taxa between the ETEC and ETEC_KR32 groups (C); relative abundance of microbial species at the species level; only taxa with >1% relative abundance are displayed (D); spearman correlation heatmap between piglets' pathological markers and differential microbiota species (E). Correlations were analyzed using Spearman's rank correlation. PCoA, principal coordinates analysis; PLS‐DA, partial least squares discrimination analysis.

### Engineered WB800_KR32 shape serum metabolites in ETEC K88‐infected piglets

We identified 2072 metabolites in the positive ion model and 1508 metabolites in the negative ion model. The main metabolites were organic nitrogen compounds and organic acids in positive ion models (Figure 4A), while organoheterocycllic compounds and fatty acyls in negative ion models (Figure S3A). The top 20 significantly differential metabolites were presented in Figure 4B and Figure S3B. The analysis of OPLS‐DA and PLS‐DA revealed that these metabolites were well distinguished (Figure 4C,D, Figure S3C,D). The heatmap of differential expression metabolites was shown in Figure 4E and Figure S3E. The analysis of the KEGG pathway of differential metabolites has found that the pathway of phenylalanine metabolism, phenylalanine, tryptophan and tryptophan metabolism, glutathione metabolism were enriched in positive ion model (Figure 4F), and histidine metabolism, pamothenate and the pathway of CoA biosynthesis, selenocompound metabolism, and pyruvate metabolism were enriched in negative ion model (Figure S3F).

**Figure 4:**
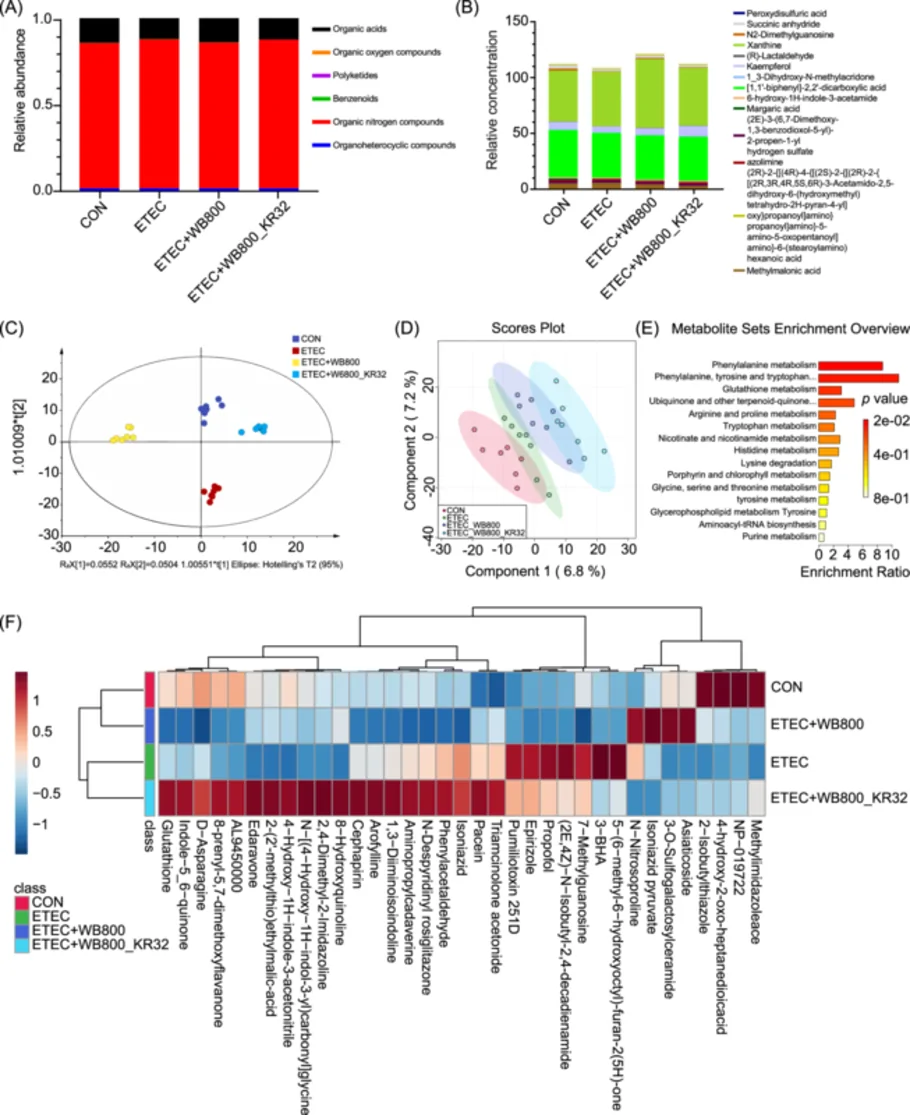
WB800_KR32 reshapes serum metabolome in enterotoxigenic *Escherichia coli* (ETEC) K88‐infected piglets (positive ion mode) (*n* = 7). Relative abundance of major metabolite classes across treatment groups (A); the effect of WB800_KR32 on top 20 metabolites in ETEC K88 infected piglets (B); the OPLS‐DA score plots showing metabolic profile separation among groups; each point represents one piglet (C); differential metabolites defined by PLS‐DA analysis (D); heatmap of differential expression metabolites, with color intensity indicating normalized expression levels (E); KEGG pathway enrichment analysis plot, bar length indicates enrichment ratio (F). PLS‐DA, partial least squares discrimination analysis.

### Engineered WB800_KR32 regulate miRNAs profiles in ETEC K88‐infected piglets

The analysis of filtered reads quality was presented in Table S5. Overall, it meets the standard of sequencing. The results of reference genome alignment were presented in Table S6. The length of small RNA was most enriched in 16–29 nt (Figure S4A). The results of the differential miRNA expression volcano map were shown in Figure S4B,C. We obtained 192 miRNAs, including 129 novel miRNAs and 63 known miRNAs (Figure S4B). Compared with the ETEC group, there were 104 miRNAs up‐regulated and 27 miRNAs down‐regulated in the CON group (Figure S4D). Compared with the EWK (ETEC + WB800_KR32) group, there were 17 miRNAs up‐regulated and 67 miRNAs down‐regulated in the ETEC group (Figure S4D). The results of hierarchical clustering of differentially expressed miRNAs are shown in Figure 5A, the CON group and EWK group were clustered together, which suggests that their expression patterns are more similar. Furthermore, according to the miRNA expression pattern, the miRNAs whose expression tendency in the CON and EWK groups was inconsistent with the ETEC group were screened out (Table S7). The primer sequences of selected miRNAs are presented in Table S8. And verified by RT‐qPCR method, compared with the CON group, the expression level of let‐7f‐5p, let‐7d‐5p, novel‐ssc‐miR1416‐5p, and novel‐ssc‐miR1284‐3p were significantly decreased in the ETEC group and EWK group (Figure S4E–I). Interestingly, we found that compared with the ETEC group, the expression level of novel‐ssc‐miR‐1250‐5p was significantly increased in the CON and EWK group (Figure 5B). And then we performed KEGG pathway of predicted targeted genes of novel‐ssc‐miR‐1250‐5p, which were enriched in focal adhesion, tight junction, NFκB signaling pathway, and inflammatory bowel disease, et al. (Figure 5C). Among the functions of the immune system, immune disease, and signal transduction, there is a common gene, *RelA*, also named *NFκB*. We performed RT‐qPCR methods to verify its expression level; the results showed that compared with the ETEC group, the expression level of *RelA* was significantly decreased in the EWK group and showed no significant difference with the CON group (Figure 5D–F).

**Figure 5:**
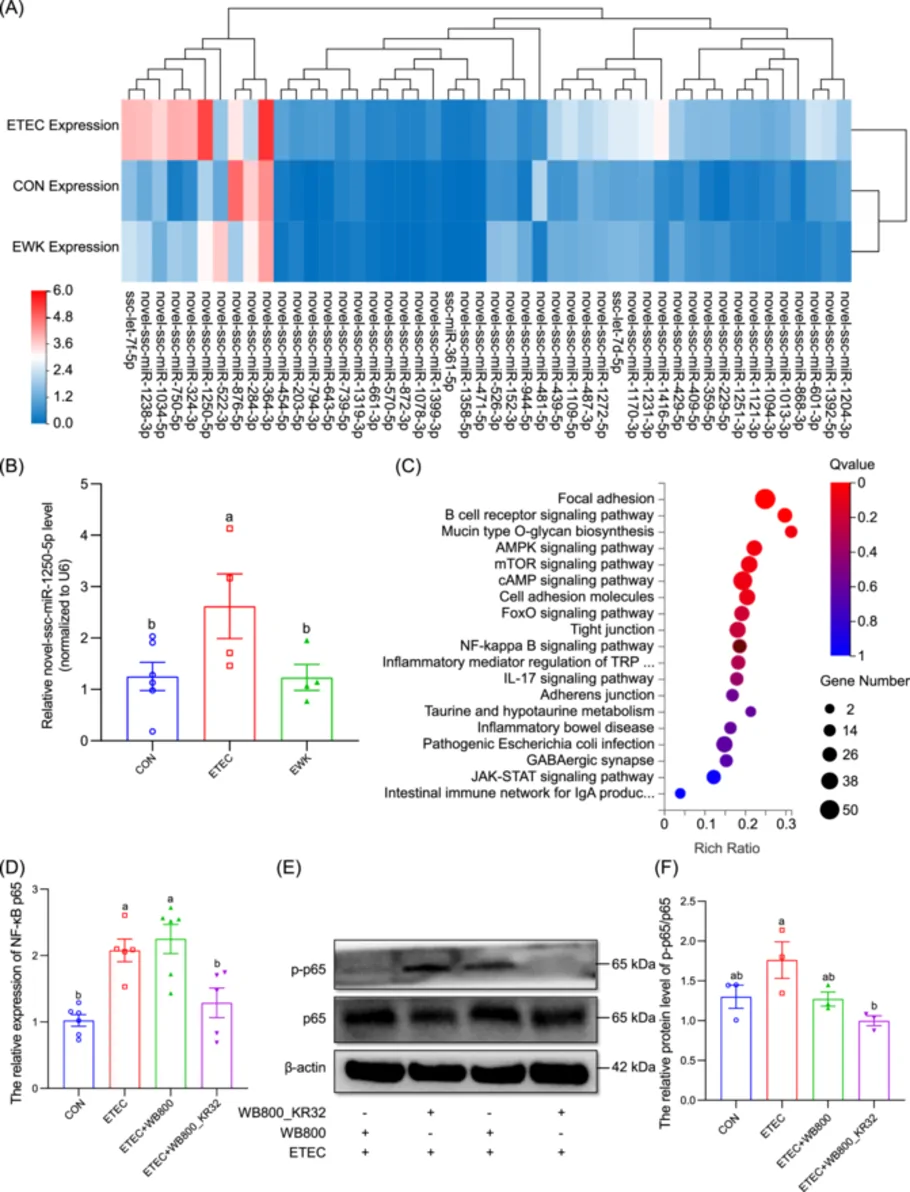
WB800_KR32 modulates ileal microRNA profiles in ETEC K88‐infected piglets (*n* = 7). Hierarchical clustering heatmap of differentially expressed miRNAs, color intensity reflects normalized expression (A); RT‐qPCR validation of novel‐ssc‐miR‐1250‐5p, normalized to U6 (B); KEGG pathway enrichment analysis of miRNA targets, bubble size indicates pathway impact, and color denotes Q value (C); the relative expression level of RelA (predicted novel‐ssc‐miR‐1250‐5p target), normalized to β‐actin (D); the western blot analysis of NF‐κB p65 protein, representative blots (E), and quantification normalized to β‐actin (F). Differences were evaluated using one‐way ANOVA with Duncan's multiple range test. ETEC, enterotoxigenic *Escherichia coli*.

### Fermented supernatant of WB800_KR32 against ETEC K88‐infection in IPEC‐J2 cells

We defined the incubation time and concentration of ETEC K88 on IPEC‐J2 cells. We found that compared with 6.2 × 10^8^ cfu/mL treatment, the cell viability was reached 62.53%, which is near 50% in 6.2 × 10^6^ cfu/mL ETEC K88 treated for 2 h. In contrast, incubation for 1 h had no significant effect on cell viability (Figure 6A). Therefore, we chose 6.2 × 10^6^ cfu/mL ETEC K88 treated for 2 h as the ETEC K88 infection model. And then we determined that 40% of the supernatant of fermented WB800_KR32, other than 40% supernatant of fermented WB800, had a protective effect on ETEC K88 infection in IPEC‐J2 cells (Figure 6B). Further, we examined the permeability of IPEC‐J2 cells treated with supernatant of fermented WB800 or WB800_KR32 and ETEC K88. The results showed that 40% supernatant of fermented WB800_KR32 could significantly decrease the activity of LDH (Figure 6C) and DAO (Figure 6D), but had no significant effect on the level of D‐LA (Figure 6E). The results of barrier function showed that 40% supernatant of fermented WB800_KR32 had no significant effect on the gene expression of *Muc1*, *Claudin‐1*, and *Occludin* (Figure 6F) but had a protective effect on the protein expression of Occludin and ZO‐1 (Figure 6G–I). The results of cytokine showed that treatment with 40% supernatant of fermented WB800_KR32 could significantly decrease the level of IL‐6, IL‐17, and sIgA, while significantly increasing the level of IL‐10 (Figure 6J–M). 40% supernatant of fermented WB800_KR32 could significantly down‐regulate the gene expression of *IL‐6*, *IL‐8*, and *NFκB p65* (Figure 6N–Q), but had no significant effect on the gene expression of *pBD‐1* (Figure 6O) and the relative protein ratio of p‐p65/p65 (Figure 6R–S).

**Figure 6:**
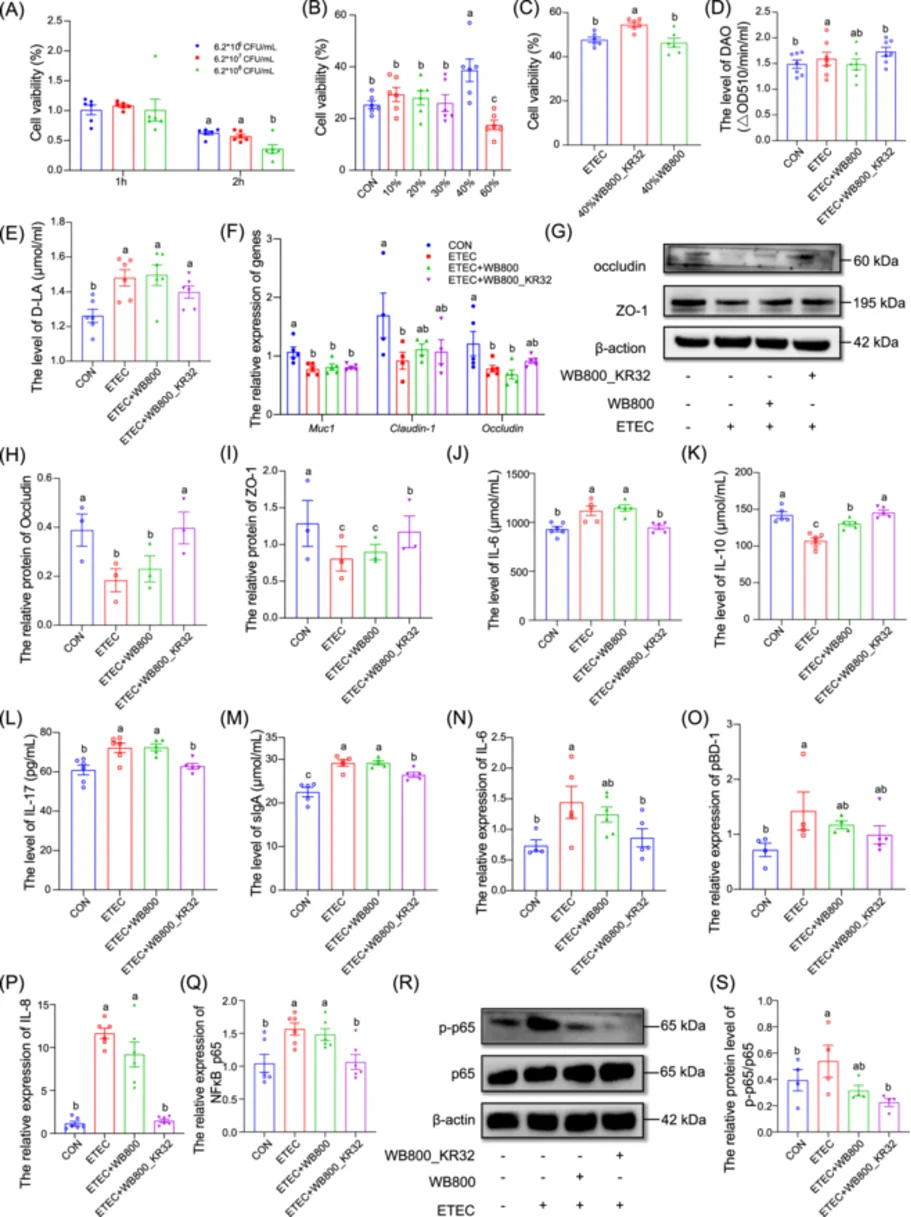
The effect of supernatant of fermented WB800_KR32 in enterotoxigenic *Escherichia coli* (ETEC) K88‐infected IPEC‐J2 cells (*n* = 4). the infected time and concentration of ETEC K88 on IPEC‐J2 cells (A); effect of treating with WB800_KR32 fermented supernatant on cell viability in ETEC‐infected IPEC‐J2 cells (B); effect of treating with WB800_KR32 fermented supernatant on cytotoxicity in ETEC K88‐infected IPEC‐J2 cells (C); effect of treating with WB800_KR32 fermented supernatant on cell permeability: DAO (D), D‐LA (E) in ETEC K88 infected IPEC‐J2 cells; effect of treating with WB800_KR32 fermented supernatant on barrier function‐related genes in ETEC K88‐infected IPEC‐J2 cells (F); effect of treating with WB800_KR32 fermented supernatant on barrier function‐related proteins in ETEC K88‐infected IPEC‐J2 cells, western blot image (G), the relative protein expression of occludin (H), the relative protein expression of ZO‐1 (I); effect of treated with WB800_KR32 fermented supernatant on cytokine, IL‐6 (J), IL‐10 (K), IL‐17 (L), sIgA (M) in ETEC K88 infected IPEC‐J2 cells; effect of treated with WB800_KR32 fermented supernatant on inflammatory immunity related genes, *IL‐6* (N), *pBD‐1* (O), *IL‐8* (P), *NFκB p65* (Q) in ETEC K88 infected IPEC‐J2 cells; the western blot analysis of NF‐κB p65 protein, representative blots (R) and quantification normalized to β‐actin (S). Differences were evaluated using one‐way ANOVA with Duncan's multiple range test. For the data shown in (A), a two‐way ANOVA was performed with infection time and drug concentration as fixed factors, followed by Duncan's multiple range test where appropriate.

## 3 DISCUSSION

The infection of *Escherichia coli* threatens the health of livestock and human beings. It's a hot research topic that applies engineered bacteria for treatment by using their capacity to produce recombinant immune‐induced components. The present research demonstrated the antimicrobial activity of the fermented WB800_KR32 supernatant using oxford cup method and LC/MS/MS analyses in vitro, the protective effect of WB800_KR32 on intestinal barrier function in ETEC K88 infected piglets, the effect of fermented WB800_KR32 supernatant on barrier function of ETEC K88 infected IPEC‐J2 cells and the mechanism of fermented WB800_KR32 supernatant prevent ETEC K88 infection induced barrier function injury via the pathway of novel‐ssc‐miR‐1250‐5p/NFκB in IPEC‐J2 cells.

Previous studies have found that AMPs KR32 have a strong antimicrobial capacity and low cytotoxicity [10]. However, there are many difficulties using the strategy of fusion expression to produce recombinant AMPs, including high cost and too many steps, which cannot be used in industry [17]. Therefore, in this study, we obtained the characteristics of targeted protein expression in engineered WB800_KR32, found that its yield and antimicrobial activity reached their highest in supernatant when fermented for 33 h. It suggested that engineered WB800_KR32, producing antimicrobial peptide KR32 a stable phase. It should be noted that the KR32 concentration detected in the fermented supernatant is below the reported MIC for ETEC. This is consistent with the engineered strain's mechanism as a “live factory,” where antibacterial activity depends on continuous local secretion rather than the bactericidal effect of free AMP alone. The present study was designed to evaluate the preventative efficacy of the live engineered bacterium, not the activity of the purified peptide. This distinction highlights the advantage of in situ AMP production by colonizing probiotics, which can achieve localized concentrations sufficient for pathogen inhibition that may not be captured in bulk supernatant measurements.

The systemic safety of the engineered WB800‐KR32 strain was previously confirmed, which revealed no adverse effects compared to controls [18]*. Bacillus subtilis* WB800, as a commonly used probiotic strain, exhibits inherent anti‐ETEC activity, likely due to its natural properties, such as competitive exclusion and production of antimicrobial substances [19]. Although WB800 shows a halo, the AMP‐expressing strain achieves a larger inhibition zone, representing a statistically significant enhancement (*p* < 0.05). This magnitude of improvement is biologically meaningful. While WB800 provides baseline protection, the AMP‐expressing strain demonstrates superior efficacy in reducing diarrhea score, improving intestinal permeability, and restoring gut barrier function more rapidly. These quantitative differences are clinically relevant.

Compared with conventional probiotics reported in literature, the engineered WB800‐KR32 strain demonstrated substantially improved efficacy. In post‐weaning ETEC models, traditional probiotic treatment increased ADG by 13.4%–17% and decreased diarrhea scores by 12%–22.5%. In our study, the engineered strain achieved a 125% ADG improvement and a 67% reduction in diarrhea score, representing a quantitatively greater response [20, 21]. This performance differential underscores the added value of AMP expression in converting baseline probiotic activity into a targeted therapeutic strategy with measurable clinical advantages.

This study demonstrated the anti‐ETEC efficacy of WB800‐KR32 at a single dose (5 × 10⁹ CFU/day); however, dose–response studies have not been conducted. Future work should systematically evaluate dose gradients to determine the minimal effective dose and safety margin, which are essential for assessing the feasibility and cost‐effectiveness of this engineered strain for commercial applications.

An integral barrier function could effectively defend against the infection of a pathogen. ETEC K88 is known to induce diarrhea and often depress the growth performance, disrupt intestinal tight junction function, increase intestinal permeability, and even threaten the life of human beings and livestock [3, 22, 23, 24]. Engineered probiotics showed the potential therapeutic activity, including cancer, obesity, and pathogen infection in animals [25, 26, 27, 28]. Our previous study has found that engineered WB800_KR32 could prevent oxidative injury of ileal mucosa via the Keap1/Nrf2 pathway in ETEC K88‐infected piglets [18]. DAO and D‐LA are the biomarkers of intestinal permeability [29]. In this study, we found that oral engineered WB800_KR32 could improve the growth performance, regulate the expression of tight junction‐related genes and protein, while decreasing diarrhea score, the level of DAO and D‐LA in serum induced by ETEC K88 infection. LDH was the biomarker of cell damage; when IPEC‐J2 cells were suffering ETEC infection, the level of LDH would significantly increase [24, 30]. The supernatant of fermented WB800_K32 could decrease the level of LDH in ETEC K88‐infected IPEC‐J2 cells. Occludin and ZO‐1 were the main components of the intestinal epithelial barrier [31, 32]. ETEC K88 infection would decrease the expression of tight junction‐related genes and proteins [33, 34]. In our study, WB800_KR32 and its fermented supernatant could significantly increase the protein expression of occludin and ZO‐1. These results suggested that engineered WB800_KR32 and its fermented supernatant showed the potential to protect the intestine against ETEC K88 infection by strengthening intestinal morphology and tight junction.

When animals are suffering stress, their immune system is activated. They will secrete cytokines to eliminate pathogens. IL‐6 will rapidly produce and regulate the acute phase response when stimulated by infection [35], it was consistent with our results. The level of IL‐6 is regulated by varied controllers, such as miRNAs [36]. IL‐10 is the key anti‐inflammatory factor that could down‐regulate the production of pro‐inflammatory factors and then protect animals from systemic inflammation [37]. IL‐17 plays a defensive role in infectious diseases, especially in pathogen infection [38]. Secretory immunoglobulin A is the first defense line that protects intestinal epithelial cells against pathogens and enterotoxin, which could modulate the microbial composition and down‐regulate pro‐inflammatory response [39]. Orally engineered WB800_KR32 could decrease the level of pro‐inflammatory cytokines while increasing the anti‐inflammatory cytokines. These results indicated that engineered WB800_KR32 could reduce intestinal immune response.

The harmony of intestinal microbiota could not only reduce the intestinal injury induced by pathogens, but also regulate the immune response, digest and ferment fibers to produce vitamins [1]. The disturbance of intestinal microbiota was considered the main reason for diarrhea of weaned piglets [1]. ETEC K88 infection significantly increases the relative abundance of *E. coli* [29, 40], it was consistent with our results. Interestingly, we found that oral WB800_KR32, not WB800, could significantly decrease the relative abundance of *E. coli*. It suggested that our engineered WB800_KR32 secreted KR32 to depress the growth of *E. coli* in vivo. The predicted KEGG pathway enrichment analysis showed that the pathways of amino acid metabolism, replication and repair, cell growth and death were enriched in the EWK group, while the pathways of infectious disease were enriched in the ETEC group. These results suggested that ETEC K88 infection caused serious injury in piglets, while oral WB800_KR32 could enhance the function of repair and nutrient utilization. The analysis of LC‐MS showed that 5‐hydroxytryptophan was significantly increased in the EWK group compared with the ETEC group. As 5‐hydroxytryptophan is the precursor of melatonin, which could decrease the bacterial load in ETEC‐infected mice while modulating the metabolism function of intestinal microbiota, such as amino acid metabolism [41]. The expression pattern of the CON group and EWK group did not consist, suggesting that WB800‐KR32 improved the imbalance induced by ETEC K88 infection.

miRNAs play a serious role in cell biology through regulating gene expression at the post‐transcriptional level. The regulation of miRNA expression was considered an important part of the host's defense against the pathogenic infection [42]. This prosperity makes miRNAs a powerful tool as therapeutics [43]. It was found that miRNAs participate in many biological processes, such as immune response [44]. It was found that pathogen infection could modulate the miRNA profile in piglets [45]. Interestingly, in our study, we found a novel miRNA named novel‐ssc‐miR‐1250‐5p. The KEGG pathway of differentially expressed miRNAs was enriched in the pathways of inflammatory response, immune response, and infectious disease. NFκB plays an important role in regulating inflammation‐related genes [46]. p65 (Rel‐A) was one of its components. The NFκB p65 signaling pathway has been a crucial point for many chronic diseases, such as inflammatory bowel disease [47]. The phosphorylate ratio of NFκB p65 was significantly increased in ETEC K88‐infected intestinal mucosa [48, 49]. NFκB p65 was one of the predicted targeted genes of novel‐ssc‐miR‐1250‐5p. Our results showed that WB800_KR32 and its fermented supernatant could significantly decrease the expression of NFκB p65 in ETEC K88‐infected piglets and IPEC‐J2 cell models. NFκB was essential for the induction of IL‐6 [50]. miR‐146b could inhibit the production of IL‐6 induced by NFκB, and then prevent inflammatory response in the normal mammary gland epithelial cell line [51]. As we all know, cytokines would damage the cellular integrity and decrease the intestinal tight junction [31].

## 4 CONCLUSION

In conclusion, engineered WB800_KR32 showed significantly enhanced antimicrobial activity in vitro. Engineered WB800_KR32 robustly regulated the barrier function through novel‐ssc‐miR‐1250‐5p/NFκB/IL‐6 pathway in ETEC K88‐infected piglets and IPEC‐J2 cells models, outperforming the parent WB800 strain, which demonstrated only modest effects. Our study provides a new perspective for applying engineered probiotics to deliver therapeutics against.

## 5 METHODS

### Bacterial strains and culture

The engineered *Bacillus subtilis* WB800/pBE2R/KR32 (WB800_KR32) and ETEC K88 were obtained in our previous study [18]. WB800_KR32 and ETEC K88 were cultured for 12 h in lysogeny broth (LB) at 37°C, 200 rpm.

### Measurement of inhibition zone diameter

The fermented supernatant of WB800_KR32 was obtained when it fermented at 24, 27, 30, 33, 36, and 39 h. The fermented supernatant of LB, both WB800 and WB800_pBE2R, was obtained (3,000 *g*, 4°C, 10 min) when it fermented at 33 h. The concentration of ampicillin sodium was 10 μg/mL. Antimicrobial activity of fermented supernatant of WB800_KR32 against ETEC K88 was tested by the Oxford cup method with some modifications [52]. Briefly, diluted inoculum (100 μL, 10^5^ CFU/mL) from the bacterial cultures was uniformly spread onto the surface of LB medium plates using sterile cotton swabs. Sterile Oxford cups (5 mm) were placed on the surface of plates poured with LB medium. Aliquots of 200 μL bacteria solution of (WB800, WB800‐pBE2R, and WB800‐pBE2R‐KR32), 10 μg/mL Ampicillin were transferred into each Oxford cup, respectively. Sterile LB medium was used as a negative control. These plates were in the refrigerator for 6 h at 4°C, then incubated at 37°C for 12 h. The inhibitory activity of WB800_KR32 against *E. coli* K88 was calculated by measuring the diameter of the transparent inhibition zone, and the antibacterial effect was evaluated by comparing inhibition zone diameters. Tests were performed in triplicate.

### LC/MS/MS analyses

LC/MS/MS analyses were performed using an Agilent 6460 triple quadrupole mass spectrometer (Agilent Technologies) equipped with an electrospray ionization source, operated in the negative ion MRM mode. Agilent Mass Hunter Workstation was used for data acquisition and processing. Nitrogen was used as the sheath gas and drying gas. The nebulizer pressure was set to 45 psi, and the flow rate of drying gas was 5 L/min. The flow rate and temperature of the sheath gas were 11 L/min and 350°C, respectively. Chromatographic separation was carried out on a Zorbax SB C18 column (150 × 2.1 mm, 3.5 µm). The HPLC mobile phases consisted of (A) 0.1% NH_3_·H_2_ and (B) Acetonitrile. The gradient program was as follows: 0–1 min, 5% of B; 1–5 min, 5%–20% of B; 5–7 min, 20%–25% of B; 7–9 min, 25%–95% of B; 9–12 min, 95% of B; the flow rate was set at 0.3 mL/min.

Mass spectrometric detection was completed by use of an electrospray ionization source in positive ion MRM mode. Detailed parameters for analyzing the targeted compounds by LC/MS/MS are listed in Table S1.

### Animal experiment and diet

Twenty‐eight Duroc × (Landrace × Yorkshire) piglets (7.09 ± 0.25 kg) weaned at 21 days were caged in individual pens and randomly allocated into four groups (*n* = 7/treatment). The piglets were fed with the basal diet, pre‐feed for 3 days, and the trial lasted 17 days. All piglets were weighed on an empty stomach at Days 1, 15, and 18. The treatments were as follows: (1) CON group: oral administration of 10 mL 0.9%NaCl (d1–d17). (2) ETEC group: oral administration of 10 mL 0.9%NaCl (d1–d14) and ETEC K88 challenge (10^9^ CFU/mL, d15–d17); (3) ETEC + WB800 group: oral administration of 10 mL (5.0 × 10^9^ CFU/mL) WB800 bacteria solution + ETEC challenge (10^9^ CFU/mL, d15–d17); (4) ETEC + WB800_KR32 group: oral administration of 10 mL (5.0 × 10^9^ CFU/mL) WB800_KR32 bacteria solution + ETEC challenge (10^9^ CFU/mL, d15–d17). The piglets were weighed at d1, d15, and d18. A corn‐soybean meal basal diet was formulated to meet the nutrient requirements according to the National Research Council [53]. All the diets used in this experiment were manufactured at Lifeng Bio‐Technology Company Ltd. facilities. All the piglets have free access to water and a diet.

The attributed score for diarrhea was as follows: 0, well‐formed feces. 1, loose feces. 2, mild diarrhea. 3, severe watery diarrhea. It was determined for each pen by a trained individual, but they had no prior knowledge of the dietary treatments provided. The score is reported as the average daily diarrhea of individual piglets [54, 55].

### Sample collection

The samples were collected at d18 for further analysis. Blood samples were collected via jugular venipuncture and centrifuged at 3,000 *g* for 10 min at 4°C. The supernatant was collected and stored at −20°C. The mucosa of the ileum, the contents of the ileum were rapidly collected and then placed into liquid nitrogen and stored at −80°C. The fresh small intestinal segment (duodenum, jejunum, and ileum; 3 cm) was collected and fixed in 4% neutral buffered formalin.

### The cytokines of serum and intestinal mucosa

The content of interleukin (IL)−6, IL‐8, IL‐10, IL‐17, and secretory immunoglobulin A (sIgA) in serum, intestinal mucosa, and supernatant of cell culture, the level of d‐lactic acid (D‐LA) and diamine oxidase (DAO) in serum and supernatant of cell culture, and the activity of lactate dehydrogenase (LDH) in supernatant of cell culture, were measured using commercially ELISA kits according to the instructions (Hunan AiFang Biological).

### Intestinal morphology analysis

The small intestinal morphology was measured by H&E staining, as previously described [56]. The villus height and crypt depth were measured by Leica Microsystem CMS GmbH using a model DM 3000 (Wetzlar, Hesse). The villus height:crypt depth ratio (V/C) was calculated. Twelve well‐oriented crypt‐villus units from each sample were measured for each group. The microvilli of the ileum were observed by transmission electron microscopy according to our previous study [57, 58].

### RNA extraction and q‐PCR

The isolation of RNA, reverse transcription, and real‐time quantitative PCR were conducted according to a previous study [57]. Total RNA was isolated using Trizol. Target genes' mRNA expression levels were calculated by the 2^−ΔΔCt^ method. β‐actin was used as the housekeeping gene. The primers of targeted genes are listed in Supplementary Table S9.

### Western blot analysis

The total protein was extracted using RIPA Lysis Buffer (Beyotime). The details method was referred to previous study [59]. The primary antibodies, including Zonula occludens protein 1 (ZO‐1), occludin, NFκB, and phosphorylated nuclear factor kappa‐B (NFκB) (ABclonal). The secondary antibody was β‐actin. The protein bands were visualized using chemiluminescent reagent (Clinx ChemiScope) and quantified using ImageJ v 1.8 software.

### Microbial analysis of ileal content

The microbial community DNA was extracted using MagPure Stool DNA KF kit B (Magen) according to the manufacturer's instructions. Variable region V3‐V4 of bacterial 16s rRNA gene was amplified with degenerate PCR primers, 341F (5'‐ACTCCTACGGGAGGCAGCAG‐3') and 806R (5'‐GGACTACHVGGGTWTCTAAT‐3'), and then sequenced using the MGISEQ. 2000 platform (BGI Genomics). The analysis of microbial data was performed according to a previous study [60]. The RAW data of ileal microbiota have been submitted to NCBI BioProject: PRJNA941041.

### Metabolites identification

In this study, the metabolites in serum were performed in Shanghai iProteome Biotechnology Co., Ltd., measured using Vanquish‐Q Exactive HF‐X (Thermo Fisher), and then identified the metabolite, matching the retention time, molecular weight, and secondary spectra with the local database. The procedure and analysis of serum metabolites were performed according to a previous study [61]. The RAW data of GC‐TOF/MS has been submitted to Metabolights NO. MTBLS7587.

### miRNAs sequencing and analysis

Total RNA was extracted from the ileal content using Trizol according to the manual instructions. The library was prepared with 1 μg total RNA for each sample. The final ligation PCR products were sequenced using the BGISEQ‐500 platform (BGI Genomics). The analysis of miRNA data was conducted according to a previous study [62]. The RAW data of miRNAs has been submitted to NCBI BioProject: PRJNA941108.

### Cell culture and treatment

The porcine small intestinal epithelial cell line (IPEC‐J2) was obtained in our laboratory [63]. The cell lines were cultured in DMEM/F12 medium containing 10% (v/v) fetal bovine serum and 1% antibiotics at 37°C and 5% CO_2_ in a moistened incubator. The cells were treated with supernatant of WB800_KR32 (fermented 33 h) for 2.5 h, and ETEC K88 (6.2 × 10^6^/mL) was incubated for 2 h.

### Statistical analysis

All results were expressed as the mean ± standard error of the mean. All data were analyzed using the one‐way analysis of variance, except for the data in Figure 6A, which were analyzed using two‐way ANOVA with infection time and drug concentration as fixed factors, followed by Duncan's multiple comparisons test using SAS 8.2 software (SAS Institute). Significant differences were declared in rows with different letters when *p* < 0.05.

## AUTHOR CONTRIBUTIONS

**Chaoyue Wen:** Conceptualization; methodology; validation; investigation; writing—original draft; writing—review and editing; visualization. **Hong Zhang:** Investigation. **Qiuping Guo:** Investigation. **Yehui Duan:** Investigation. **Sisi Chen:** Investigation. **Mengmeng Han:** Investigation. **Leli Wang:** Visualization. **Le Huang:** Formal analysis; writing—review and editing. **Yuankun Deng:** Formal analysis; writing—review and editing. **Fengna Li:** Visualization; resources; writing—review and editing. **Yizhen Wang:** Visualization; project administration; validation. **Mingliang Jin:** Conceptualization; writing—review and editing; funding acquisition. All authors have read the final manuscript and approved it for publication.

## CONFLICT OF INTEREST STATEMENT

The authors declare no conflicts of interest.

## ETHIC STATEMENT

All institutional and national guidelines for the care and use of laboratory animals were followed. All animal procedures were approved by the Committee of the Institute of Sub‐tropical Agriculture, the Chinese Academy of Sciences (No. ISA‐2022‐061).

## Supporting information


**Figure S1:** The expression pattern of the reporter plasmid.

**Figure S2:** WB800_KR32 reshapes the ileal microbiota in ETEC K88‐infected piglets.

**Figure S3:** The changes in metabolic composition in the negative ion model.

**Figure S4:** The differential expression miRNAs.

**Table S1:** MRM conditions used for LC/MS/MS analysis of analytes.
**Table S2:** Effect of oral WB800_KR32 on growth performance and diarrhea score in ETEC K88‐infected weaned piglets.
**Table S3:** Effect of oral WB800_KR32 on the cytokine of ETEC K88‐infected piglets in serum and intestinal mucosa.
**Table S4:** Effect of oral WB800_KR32 on the α‐diversity in the ileal microbiota of ETEC K88‐infected piglets.
**Table S5:** Filtered reads quality statistics.
**Table S6:** Reference genome alignment.
**Table S7:** Resistance screening of diarrhea‐related miRNAs.
**Table S8:** Partial miRNAs primer sequences.
**Table S9:** Characteristics of the primers used for real‐time PCR analysis.

## Data Availability

The RAW data of ileal microbiota have been submitted to NCBI BioProject: PRJNA941041 (http://www.ncbi.nlm.nih.gov/bioproject/941041). The RAW data of GC‐TOF/MS has been submitted to Metabolights NO. MTBLS7587 (https://www.ebi.ac.uk/metabolights/MTBLS7587). The RAW data of miRNAs has been submitted to NCBI BioProject: PRJNA941108 (http://www.ncbi.nlm.nih.gov/bioproject/941108). The data used for analyzing the article's charts and graphs has been uploaded to GitHub (https://github.com/Juanita223/ iMetaOmics7798648). Supplementary materials (figures, tables, graphical abstract, slides, videos, Chinese translated version, and update materials) may be found in the online DOI or iMeta Science http://www.imeta.science/imetaomics/.
