# Supplementary material for: Engineered Bacillus subtilis regulates intestinal barrier in ETEC K88‐infected piglets and modulates miR‐1250‐5p/NFκB pathway in IPEC‐J2
Source: IMetaOmics. 2026 May 11;3(2):e70094. doi: 10.1002/imo2.70094 (PMC13674418; doi:10.1002/imo2.70094)
Supplement: Supplementary file 1 [file IMO2-3-e70094-s001.pdf]

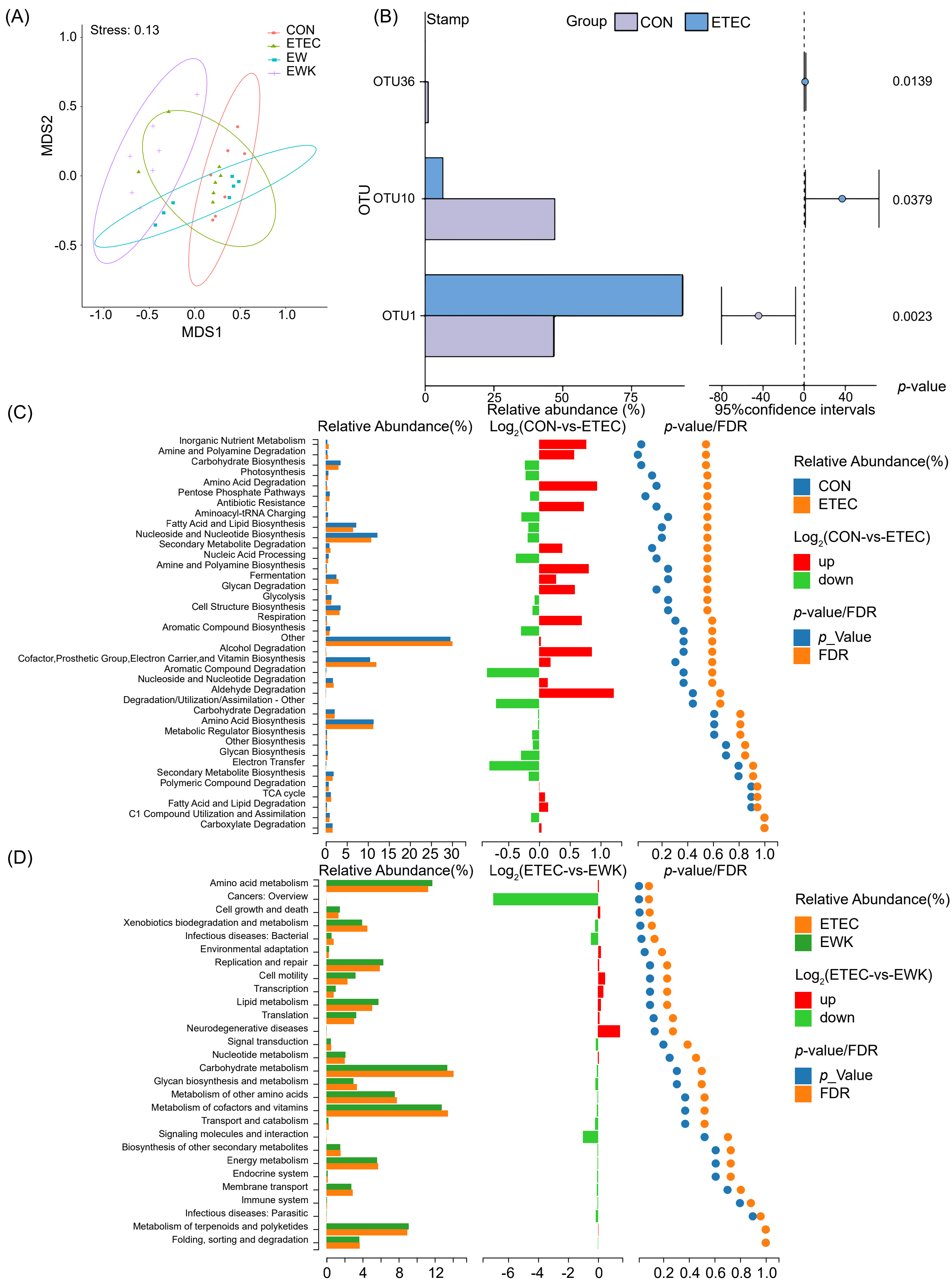

(A)
Stress: 0.13
CON
ETEC
EW
EWK
MDS2
MDS1
(B)
Stamp
Group
CON
ETEC
OTU36
OTU10
OTU1
OTU
0.0139
0.0379
0.0023
Relative abundance (%)
95%confidence intervals
p-value
(C)
Relative Abundance(%)
Log2(CON-vs-ETEC)
p-value/FDR
Inorganic Nutrient Metabolism
Amine and Polyamine Degradation
Carbohydrate Biosynthesis
Photosynthesis
Amino Acid Degradation
Pentose Phosphate Pathways
Antibiotic Resistance
Aminoacyl-tRNA Charging
Fatty Acid and Lipid Biosynthesis
Nucleoside and Nucleotide Biosynthesis
Secondary Metabolite Degradation
Nucleic Acid Processing
Amine and Polyamine Biosynthesis
Fermentation
Glycan Degradation
Glycolysis
Cell Structure Biosynthesis
Respiration
Aromatic Compound Biosynthesis
Other
Alcohol Degradation
Cofactor,Prosthetic Group,Electron Carrier,and Vitamin Biosynthesis
Aromatic Compound Degradation
Nucleoside and Nucleotide Degradation
Aldehyde Degradation
Degradation/Utilization/Assimilation - Other
Carbohydrate Degradation
Amino Acid Biosynthesis
Metabolic Regulator Biosynthesis
Other Biosynthesis
Glycan Biosynthesis
Electron Transfer
Secondary Metabolite Biosynthesis
Polymeric Compound Degradation
TCA cycle
Fatty Acid and Lipid Degradation
C1 Compound Utilization and Assimilation
Carboxylate Degradation
Relative Abundance(%)
CON
ETEC
Log2(CON-vs-ETEC)
up
down
p-value/FDR
p_Value
FDR
(D)
Relative Abundance(%)
Log2(ETEC-vs-EWK)
p-value/FDR
Amino acid metabolism
Cancers: Overview
Cell growth and death
Xenobiotics biodegradation and metabolism
Infectious diseases: Bacterial
Environmental adaptation
Replication and repair
Cell motility
Transcription
Lipid metabolism
Translation
Neurodegenerative diseases
Signal transduction
Nucleotide metabolism
Carbohydrate metabolism
Glycan biosynthesis and metabolism
Metabolism of other amino acids
Metabolism of cofactors and vitamins
Transport and catabolism
Signaling molecules and interaction
Biosynthesis of other secondary metabolites
Energy metabolism
Endocrine system
Membrane transport
Immune system
Infectious diseases: Parasitic
Metabolism of terpenoids and polyketides
Folding, sorting and degradation
Relative Abundance(%)
ETEC
EWK
Log2(ETEC-vs-EWK)
up
down
p-value/FDR
p_Value
FDR
