# Supplementary material for: Engineered Bacillus subtilis regulates intestinal barrier in ETEC K88‐infected piglets and modulates miR‐1250‐5p/NFκB pathway in IPEC‐J2
Source: IMetaOmics. 2026 May 11;3(2):e70094. doi: 10.1002/imo2.70094 (PMC13674418; doi:10.1002/imo2.70094)
Supplement: Supplementary file 2 [file IMO2-3-e70094-s002.pdf]

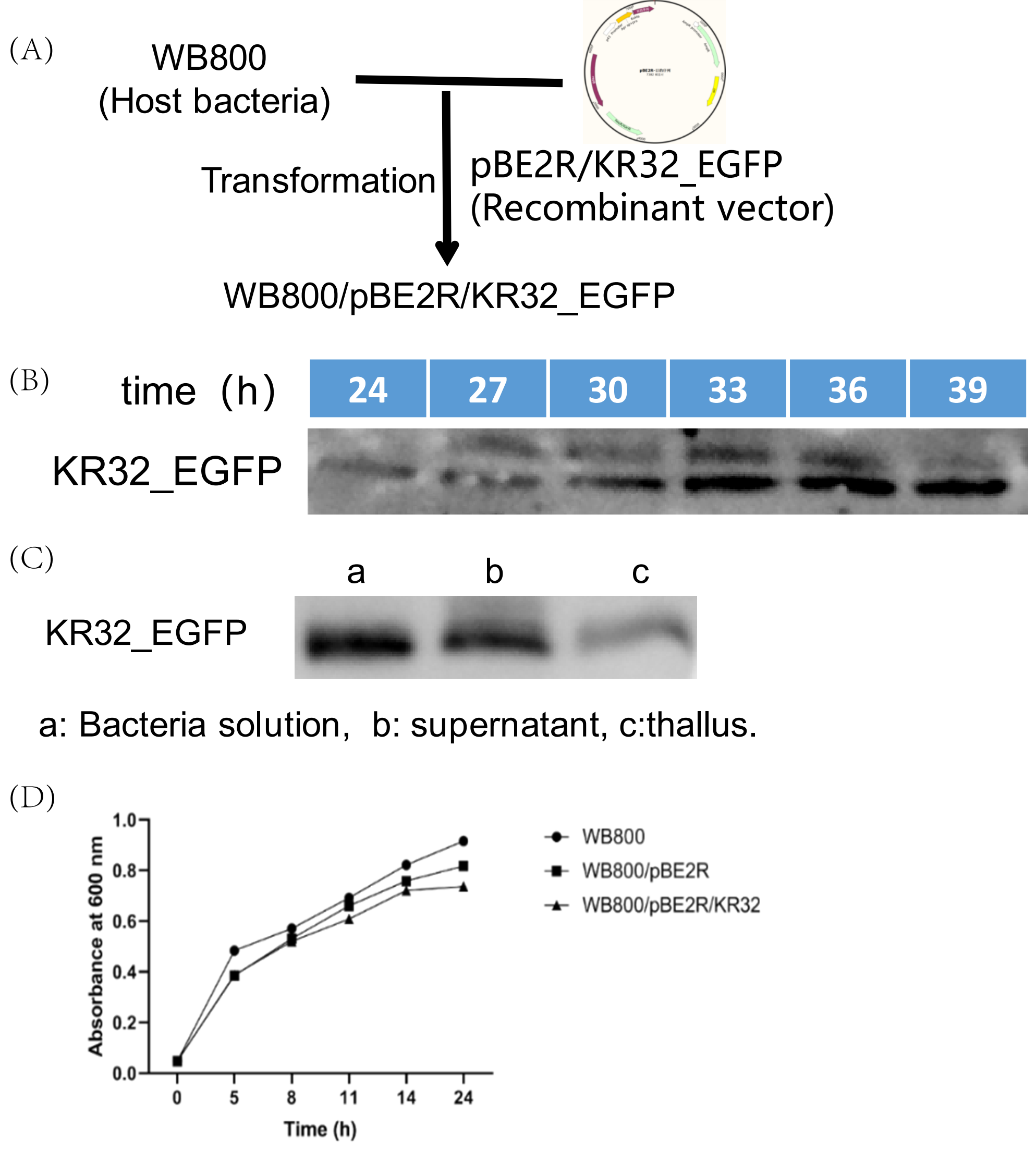
(A)
WB800
(Host bacteria)
Transformation
pBE2R/KR32_EGFP
(Recombinant vector)
WB800/pBE2R/KR32_EGFP
(B)
time (h)
24
27
30
33
36
39
KR32_EGFP
(C)
a
b
c
KR32_EGFP
a: Bacteria solution, b: supernatant, c:thallus.
(D)
Absorbance at 600 nm
Time (h)
WB800
WB800/pBE2R
WB800/pBE2R/KR32
