# Supplementary material for: Engineered Bacillus subtilis regulates intestinal barrier in ETEC K88‐infected piglets and modulates miR‐1250‐5p/NFκB pathway in IPEC‐J2
Source: IMetaOmics. 2026 May 11;3(2):e70094. doi: 10.1002/imo2.70094 (PMC13674418; doi:10.1002/imo2.70094)
Supplement: Supplementary file 3 [file IMO2-3-e70094-s003.pdf]

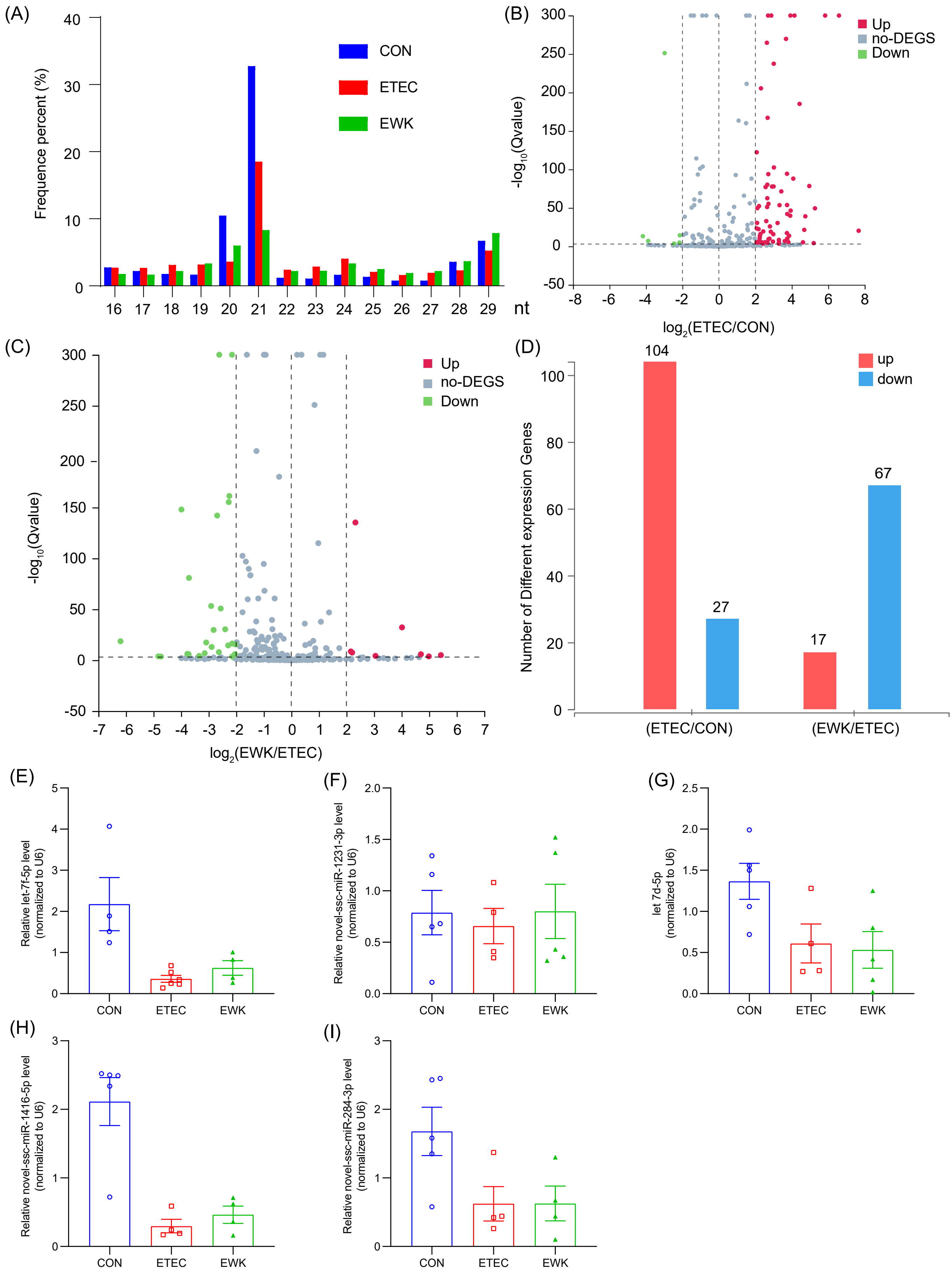
(A)
Frequence percent (%)
CON
ETEC
EWK
nt
(B)
-log10(Qvalue)
log2(ETEC/CON)
Up
no-DEGS
Down
(C)
-log10(Qvalue)
log2(EWK/ETEC)
Up
no-DEGS
Down
(D)
Number of Different expression Genes
up
down
104
27
17
67
(ETEC/CON)
(EWK/ETEC)
(E)
Relative let-7f-5p level
(normalized to U6)
CON
ETEC
EWK
(F)
Relative novel-ssc-miR-1231-3p level
(normalized to U6)
CON
ETEC
EWK
(G)
let 7d-5p
(normalized to U6)
CON
ETEC
EWK
(H)
Relative novel-ssc-miR-1416-5p level
(normalized to U6)
CON
ETEC
EWK
(I)
Relative novel-ssc-miR-284-3p level
(normalized to U6)
CON
ETEC
EWK
