# Supplementary material for: Engineered Bacillus subtilis regulates intestinal barrier in ETEC K88‐infected piglets and modulates miR‐1250‐5p/NFκB pathway in IPEC‐J2
Source: IMetaOmics. 2026 May 11;3(2):e70094. doi: 10.1002/imo2.70094 (PMC13674418; doi:10.1002/imo2.70094)
Supplement: Supplementary file 4 [file IMO2-3-e70094-s004.pdf]

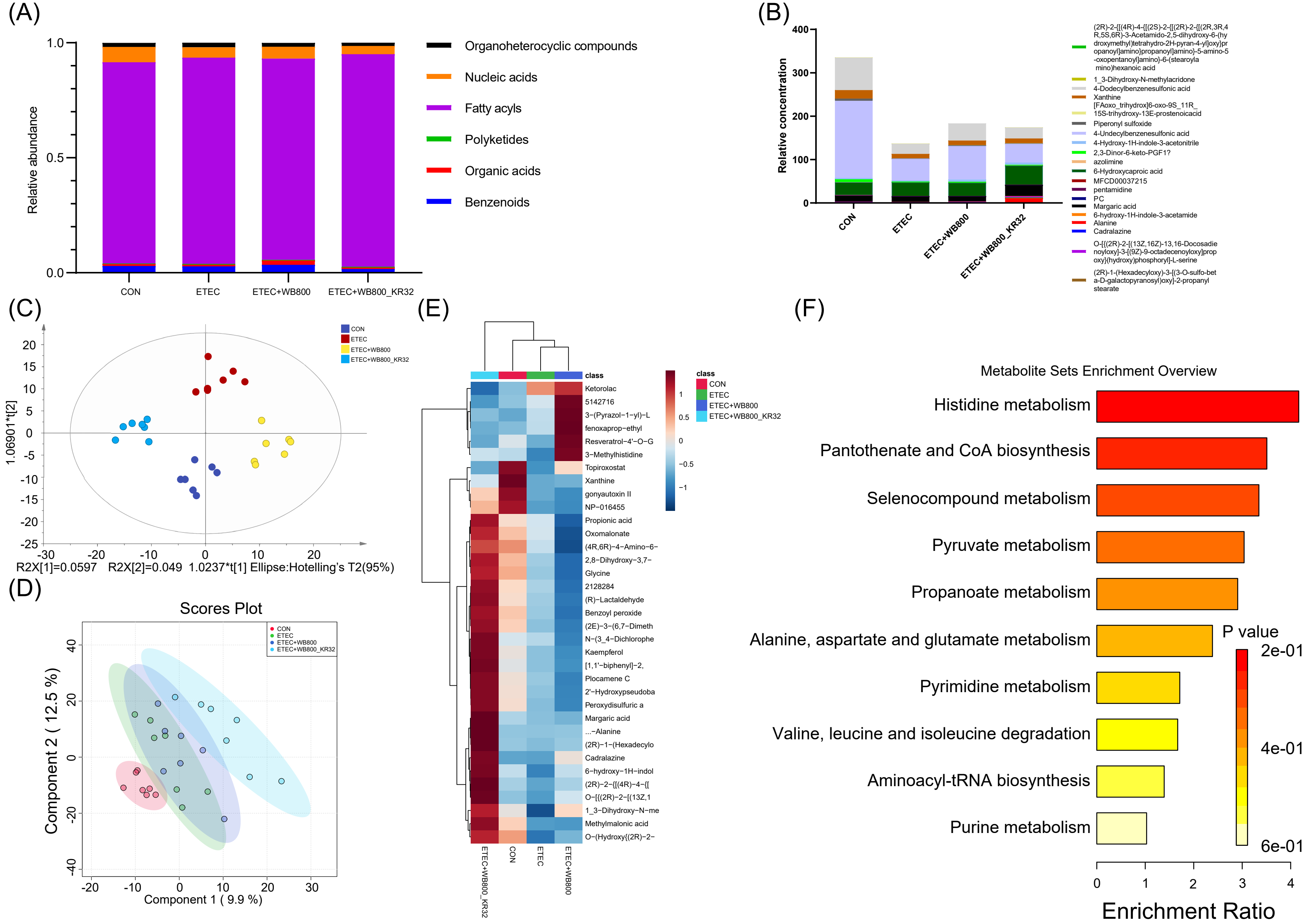
(A)
Relative abundance
Organoheterocyclic compounds
Nucleic acids
Fatty acyls
Polyketides
Organic acids
Benzenoids
CON
ETEC
ETEC+WB800
ETEC+WB800_KR32
(B)
Relative concentration
Xanthine
Piperonyl sulfoxide
4-Undecylbenzenesulfonic acid
6-Hydroxycaproic acid
Margaric acid
Alanine
Cadralazine
(C)
1.06901*t[2]
R2X[1]=0.0597 R2X[2]=0.049 1.0237*t[1] Ellipse:Hotelling's T2(95%)
(D)
Scores Plot
Component 1 (9.9 %)
Component 2 (12.5 %)
(E)
class
(F)
Metabolite Sets Enrichment Overview
Histidine metabolism
Pantothenate and CoA biosynthesis
Selenocompound metabolism
Pyruvate metabolism
Propanoate metabolism
Alanine, aspartate and glutamate metabolism
Pyrimidine metabolism
Valine, leucine and isoleucine degradation
Aminoacyl-tRNA biosynthesis
Purine metabolism
P value
Enrichment Ratio
